# SARS-CoV-2 Seroprevalence and Vaccine Uptake among Pregnant Women at First Antenatal Care Visits in Malawi

**DOI:** 10.4269/ajtmh.23-0726

**Published:** 2024-03-26

**Authors:** Lyson Tenthani, Victoria Seffren, Alinune Nathanael Kabaghe, Francis Ogollah, Monica Soko, Ruchi Yadav, Felix Kayigamba, Danielle Payne, Nellie Wadonda-Kabondo, Elizabeth Kampira, Tyson Volkmann, Nandita S. Sugandhi, Karl Seydel, Eric Rogier, Julie I. Thwing, Julie R. Gutman

**Affiliations:** ^1^ ICAP at Columbia University, International Programs – Malawi, Lilongwe, Malawi;; ^2^ Malaria Branch, Division of Parasitic Diseases and Malaria, National Center for Emerging and Zoonotic Infectious Diseases, U.S. Centers for Disease Control and Prevention, Atlanta, Georgia;; ^3^ U.S. Centers for Disease Control and Prevention, Lilongwe, Malawi;; ^4^ Blantyre Malaria Project, Kamuzu University of Health Sciences, Blantyre, Malawi;; ^5^ U.S. President’s Malaria Initiative, U.S. Centers for Disease Control and Prevention, Lilongwe, Malawi;; ^6^ ICAP at Columbia University, New York, New York

## Abstract

Many SARS-CoV-2 infections are asymptomatic, thus reported cases underestimate actual cases. To improve estimates, we conducted surveillance for SARS-CoV-2 seroprevalence among pregnant women attending their first antenatal care visit (ANC1) from June 2021 through May 2022. We administered a questionnaire to collect demographic, risk factors, and COVID-19 vaccine status information and tested dried blood spots for SARS-CoV-2 antibodies. Although <1% of ANC1 participants reported having had COVID-19, monthly SARS-CoV-2 seroprevalence increased from 15.4% (95% CI: 10.5–21.5) in June 2021 to 65.5% (95% CI: 55.5–73.7) in May 2022. Although COVID-19 vaccination was available in March 2021, uptake remained low, reaching a maximum of 9.5% (95% CI: 5.7–14.8) in May 2022. Results of ANC1 serosurveillance provided prevalence estimates helpful in understanding this population case burden that was available through self-report and national case reports. To improve vaccine uptake, efforts to address fears and misconceptions regarding COVID-19 vaccines are needed.

## INTRODUCTION

**Figure 1.**
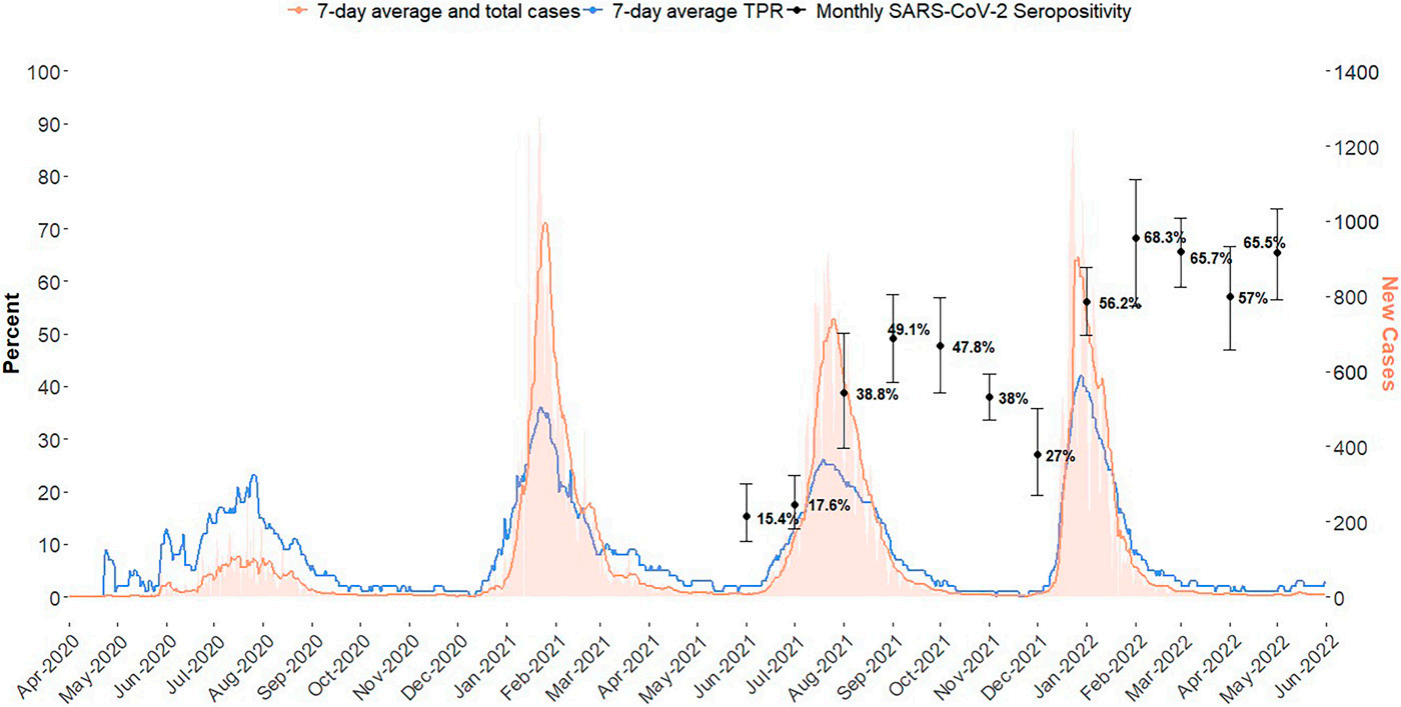
Nationally reported COVID-19 cases and test positivity rate and the monthly antenatal care visit SARS-CoV-2 seropositivity in Malawi (April 2020–May 2022). Seropositivity estimates represent either infection or hybrid immunity (infection or vaccination). Left y-axis corresponds to the seropositivity which is displayed through black points with 95% CIs as error bars. Right y-axis corresponds to case data trends, weekly new cases shown through orange bars, 7-day average shown by the orange line, and 7-day average test positivity rate shown by the blue line.

COVID-19 was declared a pandemic by the WHO on March 11, 2020; Malawi reported its first case in April 2020.^1^ Malawi, with a population of almost 20 million, had conducted 583,680 reverse transcription polymerase chain reaction (RT-PCR) or rapid tests for SARS-CoV-2 and documented 85,985 confirmed cases (test positivity rate [TPR] 14.7%) by May 31, 2022. The 7-day rolling average TPR from national case data peaks in August 2020 at 15.4%, January 2021 at 36.4%, July 2021 at 25.8%, and December 2021 at 41.6%, corresponding to the original wave and subsequent waves caused by variants of concern Alpha, Delta, and Omicron, respectively (Figure 1). By May 31, 2022, 2,640 Malawians were reported to have died of COVID-19, for a case fatality rate of 3.6%. Because testing was limited and largely targeted symptomatic individuals, case counts may have underestimated true incidence.^1^ COVID-19 vaccination began on March 11, 2021, and national roll-out was phased, starting with healthcare workers and priority groups (military, prisoners, educators, people aged over 60 years or with underlying conditions).^2^ Expansion to the general population was available by April 2021, yet uptake noticeably increased after the introduction of the COVID-19 Vaccinate Express Program in November 2021.^2^

To better understand SARS-CoV-2 prevalence, we implemented serologic surveillance among pregnant women attending their first antenatal care visit (ANC1). Women attending ANC1 have served as a sentinel surveillance population, providing proxy estimates for underlying community prevalence for other diseases, including HIV and malaria.^3^^,^^4^ In Malawi, 95% of women attend at least one ANC visit during pregnancy,^5^ providing a reliable population for surveillance. At the time surveillance began, COVID-19 vaccines were available for the general population, allowing for determination of vaccination status.

## MATERIALS AND METHODS

**Figure 2.**
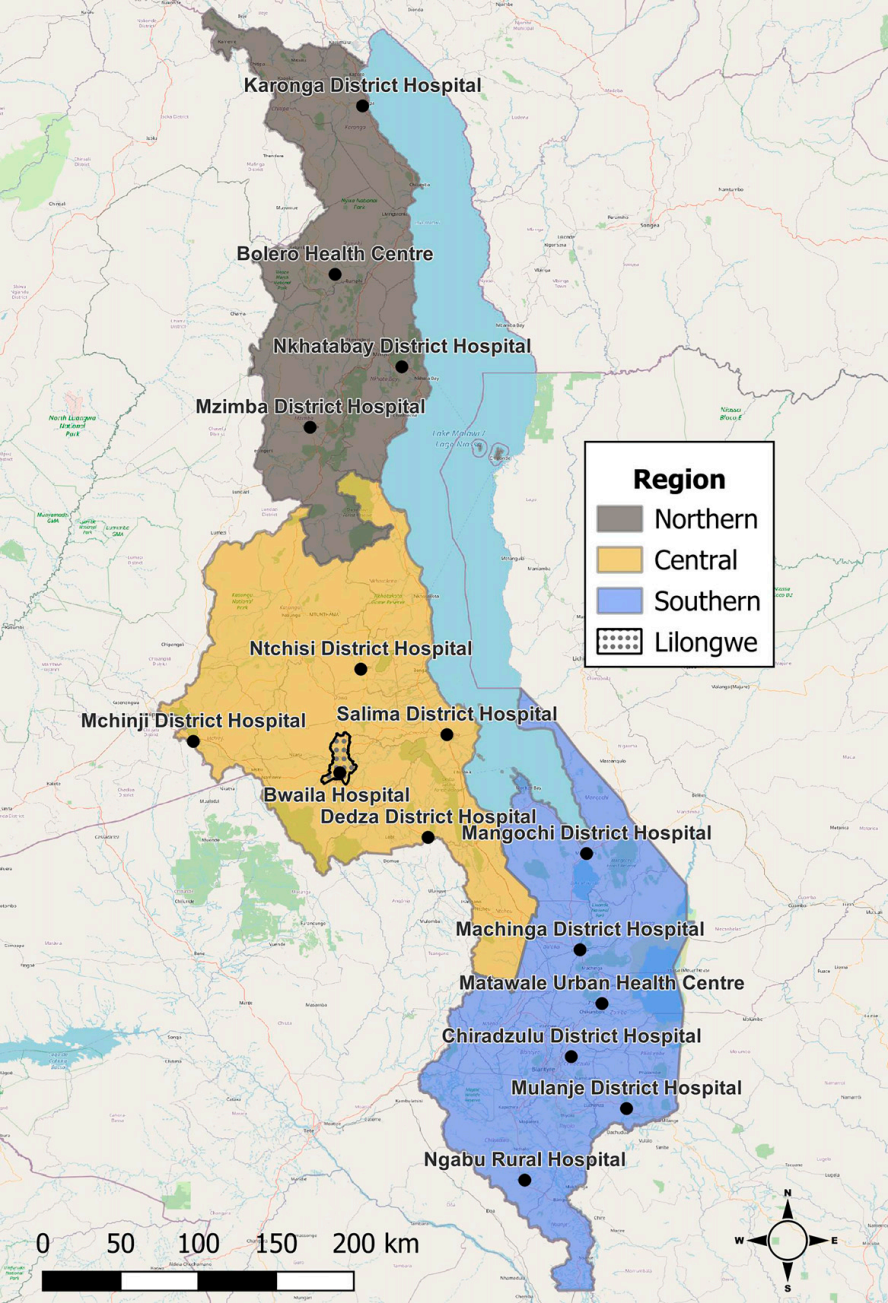
Participating health facilities in Northern, Central, and Southern Regions of Malawi. Bwaila Hospital in Lilongwe (dotted area) recruited 60 women per month, whereas all other facilities recruited 30 women per month. TPR = test positivity rate.

The protocol, approved by Malawi’s National Health Sciences Research Committee, was adapted from the WHO Unity Protocol for sero-epidemiological investigations,^6^ by omitting the age-standardized criteria. Data were collected from pregnant women attending ANC1 in 15 health facilities across Malawi from June 2021 through May 2022. Facilities were purposively selected to ensure distribution across Malawi’s three regions (Central, Northern, Southern), adequate ANC1 attendance, and reflect varying levels of district-level case burden (Figure 2). For 1 week each month, 30 ANC1 attendees from each facility provided written consent to participate (60 from the largest facility Bwaila Hospital, in the capitol Lilongwe, Central Region).

Participants were interviewed about demographics, COVID-19 risk status, prior COVID-19 exposure, COVID-19 vaccination history, and vaccine hesitancy. Vaccination status was determined by reviewing the vaccine card, or, in its absence, by participant self-report. Data were collected on tablets using ODK (OpenDataKit) software. A dried blood spot (DBS) was collected from the routine finger prick performed at ANC1 for point-of-care testing of hemoglobin, HIV, and syphilis. The DBS were tested at the Kamuzu University of the Health Sciences laboratory in Blantyre for SARS-CoV-2 antibodies. Antibody testing was performed using Tetracore’s FlexImmArray™ 7-Plex SARS-CoV-2 Human IgG Antibody Test, a multiplex bead-based assay that includes three SARS-CoV-2 target proteins (receptor-binding domain [RBD], nucleocapsid protein [N protein], and a hybrid of the two [RBD-N protein]).^7^ The manufacturer-established cut-points for signal ratios and agreement on all target proteins for assigning positivity was followed. Subsequently, we grouped results into categories of infection only, infection/vaccination, indeterminate, and no detectable IgG response using anti-RBD and anti-N analyte-specific signal ratios in combination with vaccine history (Supplemental Figure 1).^8^ Questionnaire and serology data were weighted based on total monthly ANC1 attendance at participating facilities. Proportions and corresponding 95% CI were calculated with the weighted design using R version 4.2.1.^9^

## RESULTS

We enrolled 5,764 women; the median age was 24 years (interquartile range: 19–28). The most common occupations were farmer/casual laborer (39.4%; 95% CI: 20.4–62.0) or student/unemployed (41.3%; 95% CI: 23.4–62.0). A previous positive COVID-19 test was reported by 0.7% (95% CI: 0.0–2.0) of women; those from the Northern region reported a higher proportion (2.5%, 95% CI: 0.0–10.0) compared with those from Central (0.0%, 95% CI: 0.0–1.3) and Southern (0.0%, 95% CI: 0.0–1.0) regions. Only 1.0% (95% CI: 0.0–2.0) reported direct contact with a household member who had had a presumed or confirmed COVID-19 infection.

**Figure 3.**
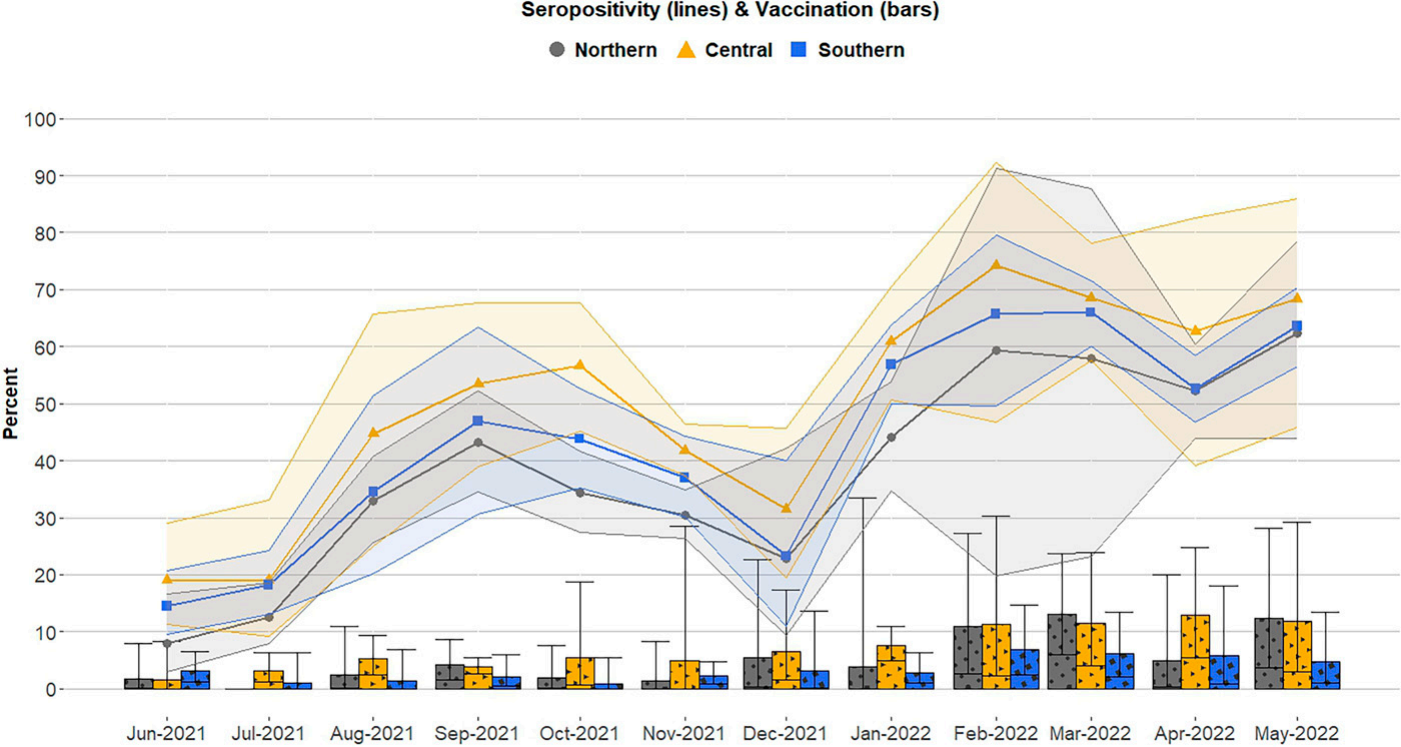
Monthly antenatal care SARS-CoV-2 seropositivity and vaccination by region, June 2021–May 2022. Trend lines with symbol markers show the regional seropositivity estimates shaded by respective 95% CIs. Bars in corresponding colors represent the proportion vaccinated with respective 95% CIs as error bars.

Seropositivity peaked at 68.3% (95% CI: 55.4–79.4) in February 2022 and lagged slightly behind peaks in national case data (Figure 1). Seropositivity ranged from 15.4% (95% CI: 10.5–21.5) to 68.3% (95% CI: 55.4–79.4) during the study period; the nadir was in June 2021, the first month of data collection, with seroprevalence ranging from 8.0% (95% CI: 2.9–16.6) in the Northern Region to 19.0% (95% CI: 11.3–29.1) in the Central Region (Figure 3). Regional seropositivity was highest in February 2022, at 59.4% (95% CI: 19.8–91.3) in the Northern and 74.2% (95% CI: 46.7–92.3) in Central (Figure 3) Regions.

Overall, 355 (5.4%; 95% CI: 4.0–7.0) participants were vaccinated, of these, vaccination was confirmed by vaccination card for 73%. In June 2021, 2.1% (95%CI: 0.8–4.4) reported vaccination, increasing to 9.5% (95% CI: 5.7–14.8) by May 2022. All but two of the vaccinated participants reported adenoviral vector vaccines, Oxford/AstraZeneca (61.1%; 95% CI: 49.5–72.0) or Janssen/J&J (37.7%; 95% CI: 26.8–50.0). Of anti-RBD seropositive women (*N* = 3,055), 4.1% (95% CI: 3.0–6.0) reported COVID-19 vaccination and were therefore classified as having seropositivity due to either vaccination or hybrid immunity. Women with no vaccine history but both anti-RBD and anti-N positivity were classified as infection-only and account for 46.8% (95% CI: 39.8–54.0) of the total sample. Of the remaining women, 44.3% (95% CI: 37.9–51.0) no detectable IgG or indeterminate results (4.8%; 95% CI: 4.1–6.0).

Among unvaccinated individuals asked about reasons for not getting vaccinated (starting November 2021, multiple option responses were allowed; *N* = 3,092), the most common responses included fear of death (42.3%; 95% CI: 30.4–55.0) and fear of pregnancy complications (27.4%; 95% CI: 16.0–43.0); 25.0% (95% CI: 15.8–37.0) were concerned about infertility, and 22.9% (95% CI: 12.3–38.0) mentioned concerns regarding side effects. Religious reasons for hesitancy were reported by 13.3% (95% CI: 6.6–25.0). Only 5.9% (95% CI: 3.9–9.0) of women reported that they did not have access to the vaccine, and 2.4% (95% CI: 0.8–7.0) reported they were not worried about catching COVID-19.

Throughout the surveillance period, an increasing number of ANC1 attendees had detectable antibodies, highlighting the increasing exposure to SARS-CoV-2 and vaccination over time. With less than 1% of our surveyed population reporting prior COVID-19 test positivity, we hypothesize that national case data underestimates the true burden of infection. In 2020, Theu et al.^1^ found a similar trend in five districts of Malawi, where active case detection in the community identified higher case burden than was reported passively through national statistics. This highlights the need for multiple surveillance approaches that provide complementary prevalence estimates to fill gaps in routine reporting.

Seropositivity peaked after epidemic waves and caused by variants of concern. We noted a decline in seropositivity following the Delta wave (June–August 2021), with a smaller decline following the subsequent Omicron wave (December 2021–February 2022). These trends held across the three regions. Although the surveillance period after the waves is not comparable, the difference may be explained by improvements in vaccination rates after Omicron. As vaccination increased in late 2021, a greater proportion of seroprevalence can be attributed to both vaccination and hybrid immunity.^10^^–^^12^ Although seroprevalence estimates follow patterns of virus circulation, cross-sectional estimates limit our ability to discuss timelines or infection correlates for antibody waning. Similar wave-specific peaks in seroprevalence were reported in Malawi in October 2020 (18.5%) and May 2021 (64.9%) among 5,085 national blood donor samples.^13^ The substantially higher proportion among blood donors in May 2021 compared with our population in June 2021 may reflect that blood donors represent a more urban population at higher risk of exposure.

Vaccines became available in Malawi in March 2021; however, vaccination uptake remained low throughout 2021. In November 2021, the Ministry of Health aimed to make vaccination more accessible by implementing mobile vaccination campaigns. Notable increases in vaccination in our sample followed, whereby 9.5% (95% CI: 5.7–14.8) were vaccinated in the last month of surveillance. As of May 2023, 26% of the Malawian population is reported to be vaccinated, which is still well below vaccination rates in other countries.^14^ Fear of side effects, including infertility, death, and pregnancy complications, were the most reported barriers to vaccine uptake. Increased education campaigns and health worker sensitization to combat COVID-19 vaccination misinformation alongside targeted approaches to reach pregnant women should be considered.^15^

This study included a couple of limitations: first, all participants were recruited over a single week each month, causing seroprevalence estimates to be aggregated to the week of visit. This does not allow for investigating the temporal relationship between national case data and serological data, as has been illustrated by others.^16^^,^^17^ Second, although ANC1 attendees have provided sentinel estimates for population prevalence for other diseases, this has yet to be conclusively demonstrated for SARS-CoV-2. We encourage the comparison of our findings to population-level serology estimates to improve understandings of this relationship for COVID-19.

## CONCLUSION

This study demonstrates the utility of the ANC platform for rapid introduction of surveillance for a novel disease prevalent in an adult population and tracking uptake of instituted mitigation measures.

## Supplemental Materials

10.4269/ajtmh.23-0726 Supplemental Materials
