# Supplementary figures and images for: SARS-CoV-2 Seroprevalence and Vaccine Uptake among Pregnant Women at First Antenatal Care Visits in Malawi
Source: Am J Trop Med Hyg. 2024 Mar 26;110(5):989–93. doi: 10.4269/ajtmh.23-0726 (PMC11066362; doi:10.4269/ajtmh.23-0726)

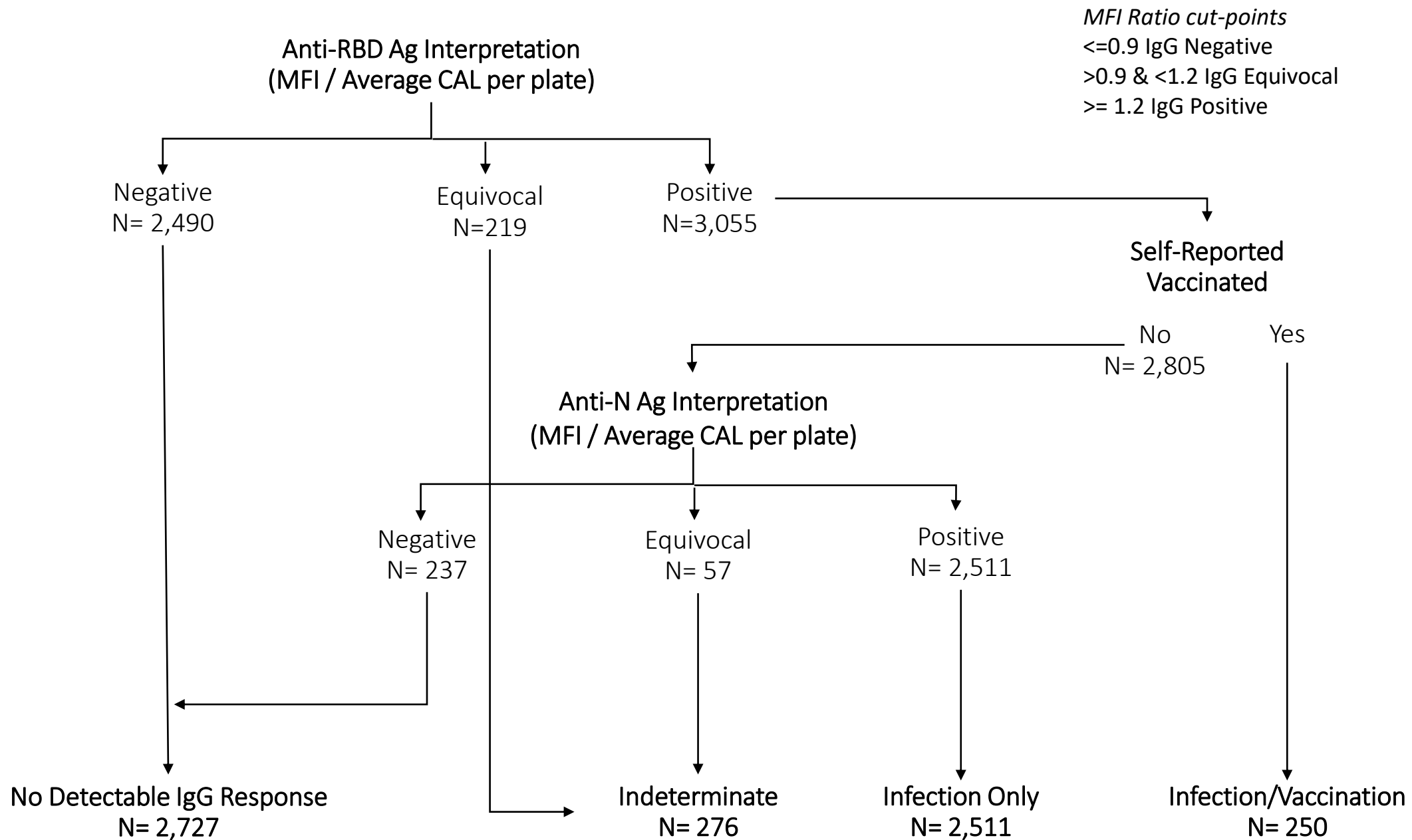

Supplement: Supplemental Materials [file tpmd230726.SD1.pdf]
